# Polygenic risk score-guided personalized osteoporosis screening: a population-based study

**DOI:** 10.1186/s12916-025-04601-1

**Published:** 2026-01-14

**Authors:** Dongxue Wang, Wen Sun, Di Liu, Huan Yi, Xiao Wang, Xiaodan Li, Jianguang Ji

**Affiliations:** 1 Faculty of Health Sciences, University of Macau, Taipa, Macau SAR, China; 2 Department of Gynecologic Oncology, Fujian Maternity and Child Health Hospital College of Clinical Medicine for Obstetrics & Gynecology and Pediatrics, Fujian Medical University, Fuzhou, 350001 Fujian China; 3 National Key Gynecology Clinical Specialty Construction Institution of China, Fujian Provincial Key Gynecology Clinical Specialty, Fujian Maternity and Child Health Hospital, Affiliated Hospital of Fujian Medical University, Fuzhou, China; 4 Center for Primary Health Care Research, Lund University, Lund, Sweden; 5 Department of Nursing, Peking University People’s Hospital, Beijing, China

**Keywords:** Osteoporosis, Polygenic risk score, Screening age, Women health

## Abstract

**Background:**

Despite robust evidence that genetic factors substantially influence both osteoporosis development and fracture risk, current screening guidelines fail to incorporate genetic factors into risk-assessment protocols. This study aims to determine personalized screening ages for osteoporosis based on polygenic risk score (PRS).

**Methods:**

This prospective cohort study utilized data from 223,818 women in the UK Biobank, and only participants who were osteoporosis-free at baseline were included in the study. Participants were categorized into three subgroups (low, medium, and high groups) based on their PRS. Ten-year cumulative risk of osteoporosis, risk-adapted starting age of osteoporosis screening, and risk advancement periods (RAP) of women across different stratifications based on PRS were calculated as the main outcomes.

**Results:**

In the general female population at age 65 (current US Preventive Services Task Force recommended screening age), the 10-year cumulative osteoporosis risk was 5.95%. Women reached this risk threshold depending on their PRS, which was 60 years for high-risk women and 69 years for low-risk women. Compared to medium-risk participants, high-risk participants developed osteoporosis 4.99 years earlier (*RAP*, 4.99; 95% *CI*, 4.94, 6.28), whereas low-risk participants developed it 4.89 years later (*RAP*, − 4.89; 95% *CI*, − 6.54, − 4.69).

**Conclusions:**

The integration of PRS could revolutionize osteoporosis prevention by enabling early detection in genetically high-risk women, potentially years before the current screening guidelines. Our findings advance the field of precision prevention for osteoporosis and may significantly reduce the population burden of osteoporotic fractures.

**Supplementary Information:**

The online version contains supplementary material available at 10.1186/s12916-025-04601-1.

## Background

Osteoporosis is characterized by low bone mass and disruption of bone architecture, resulting in compromised bone strength and increased fracture risk [1]. Osteoporosis is often considered a silent disease, as it typically presents no symptoms until the first fracture occurs [2]. Osteoporotic fractures are associated with psychological distress, subsequent fractures, loss of independence, reduced ability to perform activities of daily living, and death. Evidence shows that only 40% to 60% of individuals who experience a hip fracture regain their pre-fracture mobility and ability to perform activities of daily living [3, 4]. Globally, about 8.9 million osteoporotic fractures occur annually. Hip fractures carry a particularly high burden, with an estimated mortality rate of 20–24% within 1 year of the injury [5].

Postmenopausal women face a significantly higher risk of osteoporosis and subsequent osteoporotic fractures compared to other populations, which is linked to hormonal changes during menopause, particularly after estrogen levels drop. However, the burden of osteoporotic fractures in postmenopausal women could be prevented through early detection, making them one of the most preventable fractures. The Screening for Osteoporosis in Older Women for the Prevention of Fracture (SCOOP) study in the United Kingdom (UK) suggests that screening led to a 28% reduction in hip fractures over 5 years among women aged 70 to 85 years [6, 7]. In light of both the demonstrated benefits of osteoporosis screening and the rising global incidence of osteoporotic fractures, the US Preventive Services Task Force (USPSTF) updated its guidelines in 2025 to recommend universal osteoporosis screening for all women aged 65 years and older [8].

Genetic factors have been reported to play an important role in osteoporosis and osteoporotic fractures [9, 10]. Genome-wide association studies (GWASs) have transformed osteoporosis research, identifying over 500 genetic loci associated with bone mineral density (BMD, a key indicator for assessing osteoporosis severity) and fracture risk [11–13]. Studies have shown that current risk factor-based assessment models, even the most advanced ones, can only explain 35% of the variation in BMD, leaving 65% of the variation unexplained or unpredictable by these models [14]. Evidence suggests that up to 80% of BMD variability is attributed to genetic factors [15], while the cumulative effect of genetic risk on osteoporosis can be quantified using a polygenic risk score (PRS) [16–18]. Considering the predominant role of genetic factors in osteoporosis and osteoporotic fractures, screening for osteoporosis with consideration of genetic factors might provide an individualized screening strategy that could benefit more individuals suffering from osteoporosis and osteoporotic fractures [18, 19].

The current USPSTF guidelines do not incorporate genetic risk factors for osteoporosis screening, which is a significant limitation we seek to address. Our study aims to refine screening recommendations by integrating genetic predisposition into the determination of optimal screening initiation age. By accessing the UK Biobank cohort, we will identify women at high genetic risk for osteoporosis based on their PRS who may benefit from earlier screening than the current standard recommendation of age 65. Our approach aims to advance osteoporosis prevention from population-based guidelines to precision screening tailored to individual genetic risk profiles.

## Methods

### Study participants

Data were obtained from the UK Biobank, a large-scale population-based cohort with over 500,000 individuals aged 37–73 years who were recruited between 2006 and 2010. At the assessment center, participants reported their demographics, socioeconomic status, and lifestyle factors. In addition, they underwent physical examinations and consented to being followed through record linkage. Further information regarding this cohort can be found at http://www.ukbiobank.ac.uk/.

After excluding males and females who met the following criteria: (1) genetic data did not meet quality control standards (including participants identified as outliers in heterozygosity and those with sex chromosome aneuploidy, ambiguous sex information, or an excessive number of relatives), (2) missing PRS data, (3) a history of osteoporosis before enrollment, (4) bilateral oophorectomy, (5) a fragility fracture within the past 5 years, (6) were lost to follow-up, died, or censored before an osteoporosis diagnosis or (7) withdrew from the study, a total of 223,818 participants were included in our study.

### Outcome assessment

The outcome of this study was the first diagnosis of osteoporosis, which was defined according to the 10th revision of the International Classification of Diseases (ICD-10) codes M80.0, M80.9, M81.0, and M81.9, including postmenopausal osteoporosis and unspecified osteoporosis with or without pathological fracture [9, 20]. Patients with osteoporosis were identified from the National Cancer Registry, Hospital Episode Statistics (HES), and primary care data. All the participants were followed until the first osteoporosis diagnosis, date of death, loss of follow-up, or the last censoring date (October 31, 2022, for England; August 31, 2022, for Scotland; and May 31, 2022, for Wales), whichever came first.

### Polygenic risk score (PRS)

The UK Biobank’s genotyping process and quality control have been documented elsewhere [21]. The PRS for osteoporosis used in this study was obtained from the UK Biobank PRS Release within the UK Biobank’s Research Access Platform (March 2024 version). Bayesian analysis was used to generate the score based on meta-analyses of summary statistics from external GWASs (standard PRS). Calculation of PRS was conducted by multiplying the genome-wide sum of the per-variant posterior effect size by allele dosage. Detailed information regarding the methods is available via https://biobank.ctsu.ox.ac.uk/ukb/refer.cgi?id=5202.

### Statistical analysis

#### Baseline characteristics description

For baseline characteristics, continuous variables are presented as median (interquartile range (IQR)) and categorical variables as frequency (percentage). 

#### Hazard ratio (HR) estimation and risk stratification analysis

We estimated hazard ratios (HRs) for osteoporosis using Cox proportional hazards regression, parameterized as the change in risk per one-standard deviation increase in the PRS. Model 1 was adjusted for baseline age and the first 10 genetic principal components. Model 2 further adjusted for ethnicity, body mass index (BMI), smoking status, alcohol consumption, physical activity, history of hormone replacement therapy (HRT) use, Townsend deprivation index (TDI), menopausal status, and comorbidity (details being presented in Additional File 1) [22–24]. The missing values were imputed using multiple interpolation methods.

Subsequently, we stratified participants into three risk subgroups (low risk, medium risk, and high risk) based on PRS quartiles, with cutoffs defined as follows: low risk (lowest quartile), medium risk (middle two quartiles), and high risk (highest quartile). Specifically, using the 10-year cumulative risk in the general population at age 65 as a benchmark, participants in Q2 and Q3 showing a similar risk to the general population were classified into the medium-risk group, participants in Q1 with lower risk than the general population were classified into the low-risk group, and participants in Q4 were classified into the high-risk group (Additional File 2: Fig. S1).

#### Estimating the risk-adapted starting age of screening

**Fig. 1:**
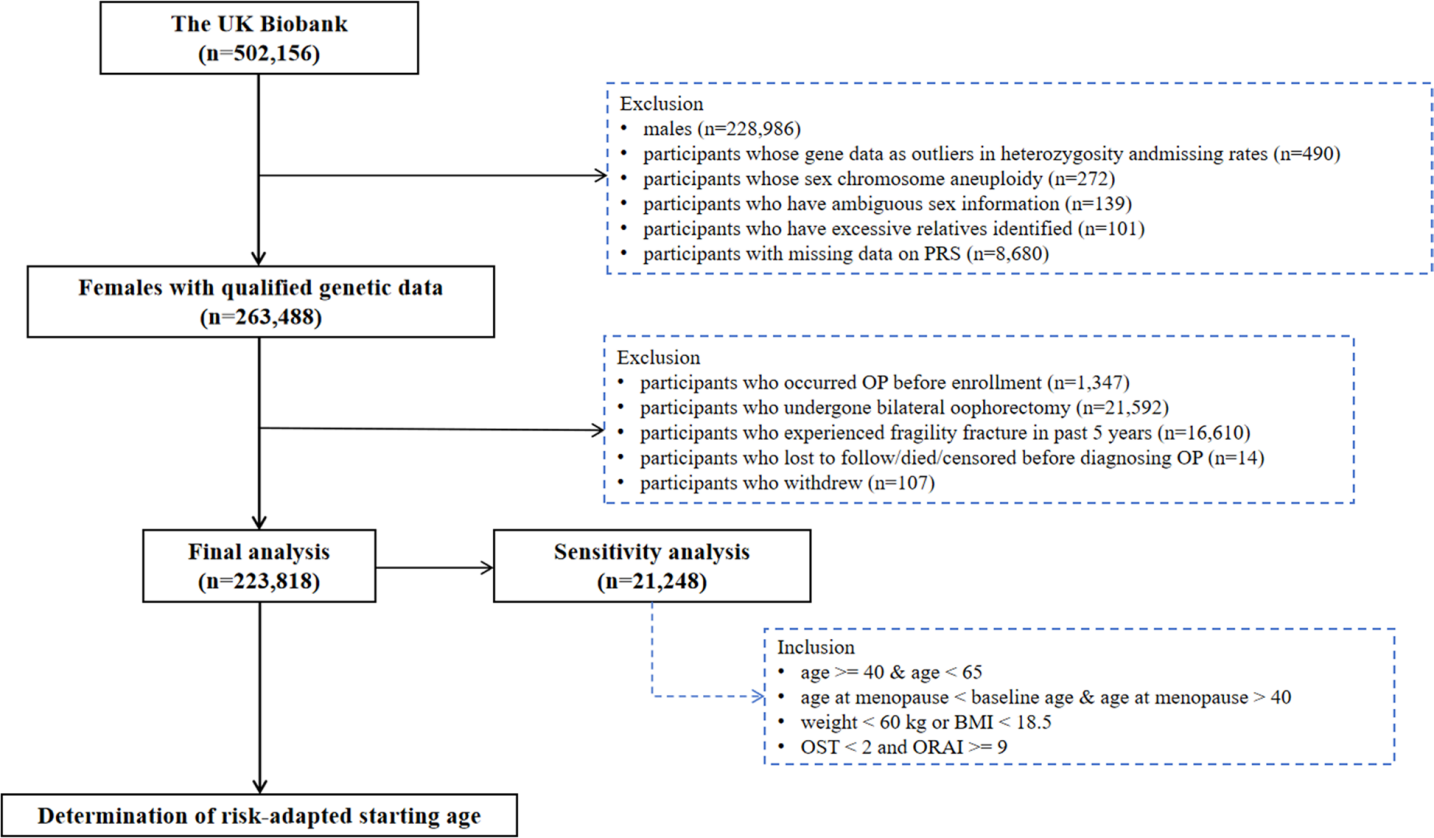
Flowchart of study design

Figure 1 presents the flowchart of the study design. Briefly, we first calculated the 10-year cumulative risk of osteoporosis for women aged 65 and used this value as the benchmark cutoff for initiating screening. Based on the current benchmark reference value, we estimated the risk-adapted starting age of osteoporosis screening for each risk group. Specifically, we defined the risk-adapted starting age as the age at which the subsequent 10-year cumulative risk in a given group reached or approximated the corresponding benchmark risk level derived from current guidelines (the 10-year cumulative risk of osteoporosis for women at age 65). The 10-year cumulative risk was calculated as 1–S(10), where S(10) denotes the Kaplan–Meier estimated survival probability at 10 years.

#### Risk advancement period (RAP) analysis

Using the medium-PRS group as a reference, we calculated the risk advancement period (RAP) for both low- and high-PRS groups [25]. Using a multivariable Cox proportional hazards model that included age at baseline, PRS category, the first 10 genetic principal components, ethnicity, BMI, smoking status, alcohol consumption, physical activity, history of HRT use, TDI, menopausal status, and comorbidity, we derived RAP point estimates by dividing the PRS regression coefficient by the age coefficient. The 95% CIs for RAPs were calculated based on the bootstrap method, with 500 stratified replicates.

#### Sensitivity analysis

We conducted several sensitivity analyses, including the following:

1. Accounting for loss of follow-up and death as competing risks, we estimated the 10-year incidence across different age groups using competing risks regression (Fine–Gray model).
2. Repeating the main analysis excluding participants with missing covariates.
3. Conducting a sensitivity analysis in women under 65 with low body weight (USPSTF recommendation grade B) [8, 26, 27]. Participant inclusion criteria are presented in Fig. 1, and a total of 21,248 females from the UK Biobank were included in the sensitivity analyses.
4. Estimating the 10-year cumulative incidence risk and RAP for osteoporotic fracture among women of different ages.
5. Estimating the 10-year cumulative incidence risk and RAP for osteoporotic fracture among men of different ages (Definition of osteoporotic fractures being presented in Additional File 1).
6. Flexible parametric survival models [28], which allow the effect of PRS to vary over time (time-varying effect) rather than being constant, were applied to calculate the adjusted HR to verify the robustness of results.

All analyses were performed using R software, version 4.4.3.

## Results

### Participant’s characteristics

A total of 223,818 female participants were included to evaluate the associations of PRS with osteoporosis risk (Fig. 1). Over a median follow-up period of 13.62 years (*IQR*, 12.81–14.29), we observed 11,639 incident cases of osteoporosis. We compared the baseline characteristics across different PRS stratifications of the participants (Table 1).

**Table 1:** Baseline characteristics for participants across different PRS risk groups

	Overall	Low risk	Moderate risk	High risk
*N*	223,818	55,955	111,909	55,954
Osteoporotic fracture (*N*)	13,572	2496	6690	4386
Follow time	13.62	13.64	13.62	13.60
(Median [IQR])	[12.81, 14.29]	[12.90, 14.28]	[12.82, 14.29]	[12.75, 14.28]
Age	57.0	57.0	57.0	57.0
(Median [IQR])	[50.0, 63.0]	[50.0, 63.0]	[50.0, 63.0]	[50.0, 63.0]
Ethnic (%)				
White	211,769 (94.6)	52,900 (94.5)	106,345 (95.0)	52,524 (93.9)
Others	12,049 (5.4)	3055 (5.5)	5564 (5.0)	3430 (6.1)
BMI (%)				
< 18.5	1732 (0.8)	408 (0.7)	839 (0.7)	485 (0.9)
18.5–24.9	89,554 (40.0)	21,776 (38.9)	44,907 (40.1)	22,871 (40.9)
25–29.9	81,639 (36.5)	20,636 (36.9)	40,809 (36.5)	20,194 (36.1)
> = 30	50,893 (22.7)	13,135 (23.5)	25,354 (22.7)	12,404 (22.2)
Smoking (%)				
Never	134,533 (60.1)	33,661 (60.2)	67,114 (60.0)	33,758 (60.3)
Previous	69,527 (31.1)	17,407 (31.1)	34,893 (31.2)	17,227 (30.8)
Current	19,758 (8.8)	4887 (8.7)	9902 (8.8)	4969 (8.9)
Alcohol consumption (%)				
Never	15,592 (7.0)	4014 (7.2)	7589 (6.8)	3989 (7.1)
Former	9045 (4.0)	2291 (4.1)	4431 (4.0)	2323 (4.2)
Occasional	23,126 (10.3)	5955 (10.6)	11,389 (10.2)	5782 (10.3)
Moderate	121,916 (54.5)	30,303 (54.2)	61,386 (54.9)	30,227 (54.0)
Increasing risk	48,032 (21.5)	11,818 (21.1)	24,079 (21.5)	12,135 (21.7)
High risk	6107 (2.7)	1574 (2.8)	3035 (2.7)	1498 (2.7)
Comorbidity (%)				
0	81,696 (36.5)	20,183 (36.1)	40,937 (36.6)	20,576 (36.8)
1–3	131,860 (58.9)	33,172 (59.3)	65,878 (58.9)	32,810 (58.6)
> = 4	10,262 (4.6)	2600 (4.6)	5094 (4.6)	2568 (4.6)
HRT use history (%)				
Yes	75,828 (33.9)	18,762 (33.5)	37,925 (33.9)	19,141 (34.2)
No	147,990 (66.1)	37,193 (66.5)	73,984 (66.1)	36,813 (65.8)
Menopausal status (%)				
Yes	155,333 (69.4)	55,955 (69.5)	77,744 (69.5)	38,680 (69.1)
No	68,485 (30.6)	17,046 (30.5)	34,165 (30.5)	17,274 (30.9)
TDI (%)				
Q1	56,003 (25.0)	13,975 (25.0)	28,143 (25.1)	13,885 (24.8)
Q2	56,024 (25.0)	13,941 (24.9)	28,116 (25.1)	13,967 (25.0)
Q3	55,928 (25.0)	14,101 (25.2)	27,925 (25.0)	13,902 (24.8)
Q4	55,863 (25.0)	13,938 (24.9)	27,725 (24.8)	14,200 (25.4)
Physical activity	1986.0	1980.0	1982.0	2002.5
(Median [IQR])	[1039.5, 3306.0]	[1039.5, 3306.0]	[1038.8, 3304.8]	[1039.5, 3319.0]

*BMI* body mass index, *TDI* Townsend deprivation index. Participants were categorized into quartiles (Q1–Q4) of the Townsend deprivation index, with Q1 representing the least deprived group and Q4 the most deprived

### Risks stratification for osteoporosis according to PRS

**Fig. 2:**
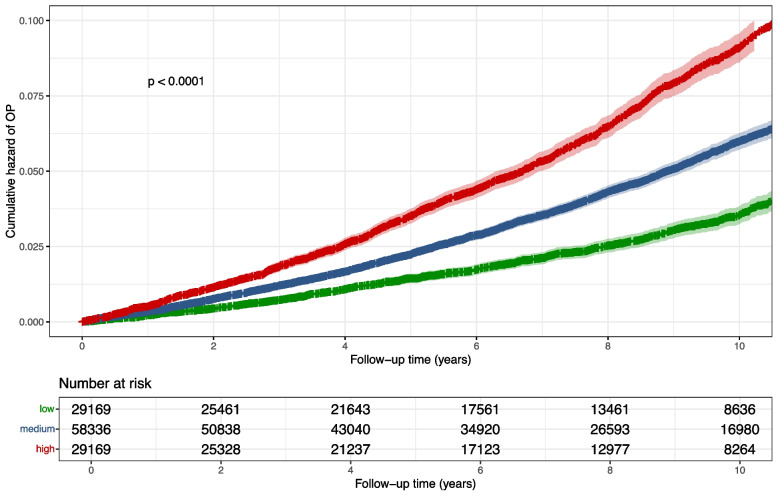
Cumulative hazard of OP in 65-year-old females across risk groups. Participants were divided into three risk groups (low, medium, and high) based on the distributions of PRS in 65-year-old female participants

Cox regression analysis revealed that each standard deviation increase in PRS was associated with a 1.40-fold higher risk of osteoporosis (*HR* = 1.40; 95% *CI*, 1.38–1.43) in women over 65 in the fully adjusted model. The risk stratification results based on the quantiles of PRS (Q1—lowest, Q2, Q3, and Q4—highest) are shown in Additional File 2: Figs. S2 -S3. After categorizing PRS components into three groups (low, medium, and high), female participants in the high-risk group exhibited approximately a 1.54-fold (95% *CI*, 1.48–1.60) higher risk of osteoporosis compared to those in the medium-risk group after adjusting for all the covariates (Table 2). Figure 2 illustrates the cumulative hazard curves of osteoporosis for three PRS-based risk groups among women aged 65 years, showing clear separation between the groups (log rank *P* < 0.0001).

**Table 2:** Risk advancement period (RAP) and risk-adapted starting age of OP screening in different risk groups based on PRS

	**Case/total (%)**	**HR**^**a**^** (95% CI)**	**HR**^**b**^** (95% CI)**	**RAP**^**b**^** (95% CI) (years)**	**Risk-adapted starting age (years) of screening**
PRS*	11,639/223,818 (5.2)	1.41 (1.38, 1.43)	1.40 (1.38, 1.43)		
Low	1848/55,955 (3.3)	0.66 (0.62, 0.69)	0.66 (0.62, 0.69)	− 4.89 (− 6.54, − 4.69)	69
Medium	5585/111,909 (5.0)	Reference	Reference	Reference	65
High	4206/55,954 (7.5)	1.55 (1.49, 1.61)	1.54 (1.48, 1.60)	4.99 (4.94, 6.28)	60

^*^Associations of genetic risk (per standard deviation increment) with OP. ^a^Model adjusted for age and first 10 genetic principal components. ^b^Model adjusted for age, ethnicity, body mass index (BMI), smoking status, alcohol consumption, physical activity, history of hormone replacement therapy (HRT) use, Townsend deprivation index (TDI), menopausal status, comorbidity, and the first 10 genetic principal components

### Risk-adapted starting age and risk-advancement period (RAP) of osteoporosis screening

**Fig. 3:**
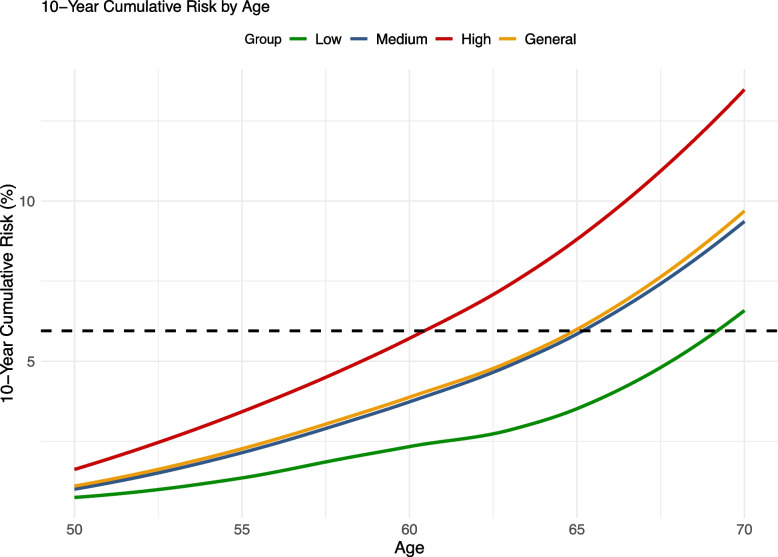
Age-specific 10-year cumulative risk curves in general female participants across risk groups. Participants were divided into three groups (low, medium, and high) based on the distributions of PRS in general female participants. The dashed line indicates the 10-year cumulative risk at the age of 65 years (the starting age of screening for women older than 65 years recommended by the US Preventive Services Task Force) in the general female participants

The 10-year cumulative risk of osteoporosis at age 65 was 5.95%, which served as the benchmark reference value. In the low, medium, and high PRS risk groups, the 10-year cumulative hazards of osteoporosis were 3.48%, 5.85%, and 8.70%, respectively (see Additional File 2: Table S1). Using this benchmark, women in the low, medium, and high PRS risk groups reached the reference risk threshold at ages 69, 65, and 60 years, respectively. Accordingly, we propose risk-adapted screening initiation ages of 69 years for low-risk individuals, 65 years for intermediate-risk individuals, and 60 years for high-risk individuals (Fig. 3).

The calculated RAP for osteoporosis screening aligned well with the risk-adapted starting ages based on the 10-year cumulative risk. Women in the high-PRS group reached an equivalent risk of osteoporosis 4.99 years earlier compared to the reference group (*RAP*, 4.99; 95% *CI*, 4.94–6.28). In contrast, women in the low-PRS group reached the same risk threshold 4.89 years later (*RAP*, − 4.89; 95% *CI*, − 6.54 to − 4.69) in the fully adjusted model (Table 2).

### Sensitivity analyses

Table S2 in Additional File 2 shows the results of age-specific 10-year cumulative risk for women over 65 across different PRS risk groups, accounting for competing risks (loss to follow-up and death). The 10-year cumulative hazards of osteoporosis were only marginally attenuated—from 3.48%, 5.85%, and 8.70% in the low, medium, and high PRS groups, respectively, to 3.38%, 5.68%, and 8.46%, while the overall risk gradient across PRS groups remained virtually unchanged. In the general population, the 10-year incidence risk for 65-year-old women (benchmark) decreased slightly from 5.95% to 5.79% after accounting for competing events. Notably, women in the high-PRS group reached a comparable risk level (5.66%) by age 60, whereas those in the low-PRS group did not reach it until age 69 (5.63%), consistent with our main analysis.

Table S3 in Additional File 2 shows the results of the sensitivity analysis for excluding the participants with missing covariates. The results were consistent with our main findings, demonstrating that our conclusions are not sensitive to the handling of missing data.

Tables S4, S5, and S6 in Additional File 2 show the results of the sensitivity analyses for postmenopausal women under 65 with low body weight. Over a median follow-up period of 13.59 years (*IQR*: 12.73–14.28), a total of 21,248 female participants from the UK Biobank were included in the sensitivity analysis, during which 1850 incident cases of osteoporosis were identified. Among postmenopausal women under 65 with low body weight, we observed similar risk patterns based on individuals’ PRS (Fig. S4 and S5 in Additional File 2). Women in the high-PRS group attained an equivalent risk of osteoporosis 5.37 years earlier than the reference group (*RAP*, 5.37; 95% *CI*, 3.73–7.06). Conversely, women in the low-PRS group reached this risk threshold 6.57 years later (*RAP*, − 6.57; 95% *CI*, − 8.87 to − 4.44) (Table S6 in Additional File 2).

The sensitivity analyses using osteoporotic fracture as the outcome are presented in Tables S7, S8, S9, and S10 of Additional File 2. The inclusion and exclusion flow of participants is shown in Additional File 2: Figs. S6 and S7. The sensitivity analyses using osteoporotic fracture as the outcome are presented in Additional File 2: Tables S7, S8, S9, and S10. The 10-year cumulative risk of osteoporotic fracture among women aged 65 was 5.22%. Comparable risks were observed in women aged 68 in the low-PRS group (5.13%) and in women aged 61 in the high-PRS group (5.25%). For men, only those in the high-PRS group reached a similar threshold, with a risk of 5.42% observed at approximately age 69. Women in the high-PRS group reached this risk 4.16 years earlier than the reference group (*RAP*, 4.16; 95% *CI*, 3.61–4.74), while those in the low-PRS group attained it 4.28 years later (*RAP*, − 4.28; 95% *CI*, − 5.04 to − 3.61) (Table S9 in Additional File 2). Similarly, men in the high-PRS group reached the risk 6.14 years earlier (*RAP*, 6.14; 95% *CI*, 4.85–7.35), whereas men in the low-PRS group reached it 6.24 years later (*RAP*, − 6.24; 95% *CI*, − 7.83 to − 4.74) (see Additional File 2: Table S10).

Table S11 in Additional File 2 presents HR and RAP estimates across PRS-based risk groups from flexible parametric survival models, consistent with the main analysis.

## Discussion

In this population-based study utilizing the UK Biobank database, we present the first evidence-based framework, to our knowledge, for optimizing osteoporosis screening ages by incorporating individual genetic risk profiles. Our findings demonstrate that women with high PRS may benefit from initiating screening at age 60, 5 years earlier than the current guidelines’ recommendation, whereas those with low genetic risk could potentially delay screening until age 69.

These personalized screening recommendations could transform osteoporosis prevention strategies, enable more efficient resource allocation, and reduce the population burden of osteoporotic fractures.

OP is a multifactorial condition influenced by both genetic and environmental risk factors. A growing body of evidence indicates that genetic factors play a significant role in the development of osteoporosis [29]. GWASs have achieved significant success in identifying the genetic predisposition to osteoporosis [30, 31]. Based on the success of the GWAS studies, we could build PRS to define the individual risk of osteoporosis. Our findings highlight the potential of using genetic risk stratification to optimize screening strategies, thereby advancing precision medicine in osteoporosis prevention. Tailoring the screening initiation based on risk factors allows high-risk individuals to be identified and monitored sooner, enabling timely intervention and reducing the probability of disease progression. The risk-adapted starting age of screening has important clinical and public health significance for individualized prevention of diseases. In current research, more attention has been paid to the risk-adapted starting age of screening regarding cancer [20, 32, 33]. A previous study based on the family history of colorectal cancer estimated that participants with different family histories reached a 10-year cumulative risk of colorectal cancer 3–29 years earlier compared to individuals at age 50 years old in the average-risk population [34]. However, there has been little exploration of the risk-adapted starting age of screening for musculoskeletal system disorders such as osteoporosis. In our study, we employed both RAP and the absolute risk of osteoporosis occurrence (10-year cumulative risk) to estimate the risk-adapted starting ages of osteoporosis screening for females based on their genetic risk profiles. We obtained similar estimates for the risk-adapted starting ages of osteoporosis screening by these two methods, which further supported our research findings.

Our study demonstrated improved performance for risk stratification and clinical implications for the derivation of risk-adapted starting ages of osteoporosis screening. This may contribute to the decision-making process for osteoporosis screening, with the potential to reduce the screening burden of people with lower risk while improving screening detection rates. Our study further evaluated the number needed to screen (NNS). The results demonstrated that the NNS to prevent one fracture was 22 in the high-risk group, which was more efficient compared with an NNS of 28 in the overall population. Importantly, the strategy of earlier screening applied to the high-PRS group prevented 1817 osteoporotic fractures in our cohort, accounting for 41.4% of all fractures that could be prevented by screening. This study represents a significant step forward in the field of personalized medicine for osteoporosis prevention. By shifting from a one-size-fits-all approach to a personalized model of care, PRS-guided screening represents a promising step toward reducing the burden of osteoporotic fractures and advancing the field of precision medicine. Cost-effective screening has been reported to depend on the prevalence of osteoporosis [35], which was highly affected by the initiation age at screening [36]. Future research can assess whether the proposed risk-based screening strategy can improve patient prognosis and save costs.

Although progress in integrating PRS into clinical practice remains limited, studies have demonstrated its significant potential for feasibility. A recent study showed that combining PRS with standardized cardiovascular risk calculators, such as QRISK2, to implement integrated risk tools (IRT) for cardiovascular diseases in primary care settings is feasible [37]. The survey revealed that the vast majority of participants (86.9%) stated they were “likely” or “very likely” to recommend the test to family or friends, 98.8% found the test helpful for personal use, and 94.6% reported that the results were easy to understand. Furthermore, healthcare providers strongly indicated that cardiovascular IRT could be easily integrated into routine primary care. Therefore, using genetic scores for population-level screening of osteoporosis holds great promise in the future. However, achieving this goal requires addressing critical challenges related to equity, infrastructure, and policy to ensure the technology is accurate, effective, and accessible to all.

### Strengths

This study has some strengths. One of the major advantages of this study is its comprehensive design, which incorporates a large, population-based cohort from the UK Biobank. Over 220,000 participants were included in this study with a median follow-up of 13 years, which allowed us to provide robust evidence on the stratification of osteoporosis risk across different genetic profiles. Additionally, the use of a Bayesian framework to calculate PRS and the subsequent derivation of risk-adapted screening ages ensures methodological rigor and reproducibility. The study also employs a validated RAP analysis and absolute risk to provide evidence for the risk-adapted starting age of osteoporosis screening, which offers a novel way to quantify how genetic risk accelerates the onset of osteoporosis. In addition, RAP and 10-year cumulative risk provided similar results for risk-adapted starting ages of osteoporosis screening, which implied the stability and reliability of the current findings.

### Limitations

Nevertheless, several limitations of this study require consideration. First, the cohort primarily consisted of white participants, which limits the generalizability of the findings to other ethnic groups. Given that genetic determinants of BMD and osteoporosis risk may vary across populations, future studies should validate the proposed screening strategy in more diverse cohorts. Second, the RAP and 10-year cumulative risk were calculated based on PRS; there are still many risk factors of osteoporosis that could be used to construct a combined risk score (i.e., blood protein, lifestyle behaviors, or comorbidities) to estimate osteoporosis screening age more comprehensively. Further research to evaluate the contribution of well-established nongenetic factors in the risk-adapted starting age of osteoporosis screening may be worth investigating. Third, the ascertainment of osteoporosis cases based solely on ICD codes from linked health records is inherently limited, as it may fail to capture individuals identified through BMD testing but without a corresponding clinical diagnosis. However, we have also explored the risk of osteoporotic fracture, which could not be affected by underdiagnosis from the linked health record. The similar results observed in osteoporotic fractures suggest that ascertainment of osteoporosis from health records may not affect our conclusions. Fourth, the study focuses on women aged 65 years or older, in line with current screening guidelines, which recommend broad screening for all women in this age group. Considering cost-effectiveness, current guidelines do not specify a recommended screening age for women under 65. Studies comparing current osteoporosis screening strategies have shown that existing guidelines (such as USPSTF, National Osteoporosis Foundation, and Canadian guidelines) exhibit low sensitivity in identifying postmenopausal women under 65 who are at risk of subsequent fractures and require BMD testing [38]. As a result, it has been challenging to establish validated screening strategies for osteoporosis in this population. Therefore, we included postmenopausal women under 65 who, according to USPSTF recommendations, may require screening as part of a sensitivity analysis to evaluate the effectiveness of PRS in risk stratification and strategy development. Although our findings suggest that genetic effects may be stronger in younger women, further research and updated algorithms are needed to develop more effective screening strategies for postmenopausal women under 65.

## Conclusions

Overall, our findings demonstrate that integrating PRS into OP screening protocols could enable personalized risk stratification, potentially improving early detection rates among high-risk women while optimizing resource allocation in preventive care.

## Supplementary Information

Additional file 1: Derivation of covariates.

Additional file 2: Figures S1–S7. Fig. S1-Age-specific 10-year cumulative risk curves in different PRS risk groups. Fig. S2 - The cumulative hazard of osteoporosis in different PRS risk groups at age 65. Fig. S3 - The cumulative hazard of osteoporosis in different PRS risk groups of all female participants. Fig. S4 - Cumulative hazard of osteoporosis in different PRS risk groups of postmenopausal women under 65 with low body weight. Fig. S5 - Age-specific 10-year cumulative risk curves in different PRS risk groups of postmenopausal women under 65 with low body weight. Fig. S6 - Flowchart of osteoporosis fracture risk as the primary outcome for women. Fig. S7 - Flowchart of osteoporosis fracture risk as the primary outcome for men. Table S1-S11. Table S1 - Age-specific 10-year cumulative risk (%) for women over 65 across different PRS risk groups. Table S2 - Age-specific 10-year cumulative risk (%) for women over 65 across different PRS risk groups, accounting for competing risks (loss to follow up and death). Table S3 - Hazard ratio (HR) and Risk advancement period (RAP) in different risk groups based on PRS (Excluding participants with missing covariates). Table S4 - Baseline characteristics for postmenopausal women under 65 with low body weight across different PRS risk groups. Table S5 - Age-specific 10-year cumulative risk (%) for postmenopausal women under 65 with low body weight across different PRS risk groups. Table S6 - Risk advancement period (RAP) and risk-adapted starting age of osteoporosis screening for postmenopausal women under 65 with low body weight across different PRS risk groups. Table S7 - Age-specific 10-year cumulative risk (%) of osteoporotic fracture for women. Table S8 - Age-specific 10-year cumulative risk (%) of osteoporotic fracture for men across different PRS risk groups. Table S9 - Risk advancement period (RAP) and risk-adapted starting age of osteoporotic fracture screening for women across different PRS risk groups. Table S10 - Risk advancement period (RAP) of osteoporotic fracture for men across different PRS risk groups. Table S9 - Risk advancement period (RAP) and risk-adapted starting age of osteoporotic fracture screening for women across different PRS risk groups. Table S10 - Risk advancement period (RAP) of osteoporotic fracture for men across different PRS risk groups. Table S11 - Risk advancement period (RAP) and risk-adapted starting age of OP screening in different risk groups based on PRS using flexible parametric survival models.

## Data Availability

Details of the data release schedule are available from https://www.ukbiobank.ac.uk/enable-your-research/apply-for-access.

## Abbreviations

PRS: Polygenic risk score
RAP: Risk advancement periods
USPSTF: US Preventive Services Task Force
GWASs: Genome-wide association studies
BMD: Bone mineral density
ICD: International Classification of Diseases
HES: Hospital Episode Statistics
SD: Standard deviation
IQR: Interquartile range
HR: Hazard ratio
OST: Osteoporosis Self-Assessment Tool
ORAI: Osteoporosis Risk Assessment Instrument
BMI: Body mass index
TDI: Townsend deprivation index
HRT: Hormone replacement therapy
IRT: Integrated risk tools
