# Supplementary material for: Polygenic risk score-guided personalized osteoporosis screening: a population-based study
Source: BMC Med. 2026 Jan 14;24:56. doi: 10.1186/s12916-025-04601-1 (PMC12849559; doi:10.1186/s12916-025-04601-1)
Supplement: Supplementary file 1 — Additional file 1: Derivation of covariates. [file 12916_2025_4601_MOESM1_ESM.docx]

## **Additional File 1**

## Derivation of covariates

**Ethnicity**

At recruitment, participants were asked in the touchscreen questionnaire to select their ethnic group among “White”, “Mixed”, “Asian or Asian British”, “Black or black British”, “Chinese”, “Other ethnic group”, “Do not know”, or “Prefer not to say”. We regrouped participants into the following ethnicity categories: ‘White, ‘Other’ and ‘Unknown’.

**The Townsend deprivation index**

The Townsend deprivation index was used as in index of socio-economic status (as a continuous variable). Based on national census output areas, this variable was previously created in the UK biobank resource. Each participant was assigned a score corresponding to the output area in which their postcode was located. Participants were categorized into quartiles (Q1–Q4) of the Townsend Deprivation Index, with Q1 representing the least deprived group and Q4 the most deprived.

**Body mass index (BMI)**

Body weight and standing height were measured at the assessment centre visit at recruitment. BMI was calculated as a participant’s body weight (kg) divided by the square of their height (m). Participants were grouped into categories of ‘< 18.5’, ‘18.5-24.9’, ‘25-29.9’, ‘≥30’ based on their BMI.

**Physical activity**

Physical activity in total metabolic equivalent task (MET) minutes per week was calculated based on a series of questions that asked about frequency and duration of walking, moderate activity, and vigorous activity.

**Smoking status**

At recruitment, participants were asked in the touchscreen questionnaire “Do you smoke tobacco now?” and “In the past, how often have you smoked tobacco?” to determine their smoking status as current, previous, or never.

**Alcohol consumption**

Participants were first asked about their drinking frequency and then, for each beverage type—red wine (glasses), white wine/champagne (glasses), beer/cider (pints), spirits (measures), fortified wine (glasses), and other alcoholic drinks (glasses)—they reported the average amount consumed per week or per month. We calculated weekly alcohol units by summing units across all beverage categories; when intake was reported monthly, values were converted to weekly units by dividing by 4.3. Units per day were derived by dividing weekly units by 7. Participants were then categorized as never drinkers, ex-drinkers, occasional drinkers (<2 units/week for both men and women), moderate drinkers (<14 units/week for both men and women), increasing-risk drinkers (14–35 units/week for women; 14–50 units/week for men), or high-risk drinkers (≥35 units/week for women; ≥50 units/week for men), in accordance with current UK drinking guidelines (Department of Health, 2016).

**Menopause status**

Female participants were considered menopausal if they met any of the following criteria: (i) self-reported menopause; or (ii) for those with unknown menopausal age, the value was imputed as 53 years, since 95% of women were reported to be mnopausal by that age.

**Hormone replacement therapy (HRT)**

At recruitment, female participants were asked in the touchscreen questionnaire “Do you regularly take any of the following medications?” to determine if hormone replacement therapy is used regularly.

**Morbidity count**

Participants reported their long-term conditions at baseline assessment. The list of morbidities described in this paper was taken from a list of 43 long-term conditions originally established for a large epidemiological study in Scotland, through systematic review, the Quality and Outcomes Framework, NHS Scotland, and an expert panel,1 and subsequently amended for UK Biobank. 20 The number of long-term conditions reported was summed and multimorbidity categorised as ‘0’, ‘1-3’ or ‘>=4’ group.

**ICD10 code of osteoporotic fracture**

M800, M8000, M8002, M8003, M8005, M8006, M8007, M8008, M8009, M801, M8019, M802, M8025, M803, M804, M8040, M8045, M8048, M8049, M805, M8050, M8055, M8057, M8058, M8059, M808, M8080, M8081, M8088, M8089, M809, M8090, M8091, M8093, M8095, M8097, M8098, M8099, S120, S1200, S1201, S121, S1210, S1211, S122, S1220, S1221, S127, S1270, S128, S1280, S129, S1290, S220, S2200, S2201, S221, S2210, S2211, S222, S2220, S223, S2230, S224, S2240, S225, S2250, S228, S2280, S229, S320, S3200, S3201, S321, S3210, S322, S3220, S323, S3230, S324, S3240, S3241, S325, S3250, S327, S3270, S3271, S328, S3280, S3281, S420, S4200, S4201, S421, S4210, S4211, S422, S4220, S4221, S423, S4230, S4231, S424, S4240, S4241, S427, S4270, S4271, S428, S4280, S429, S4290, S4291, S520, S5200, S5201, S521, S5210, S5211, S522, S5220, S5221, S523, S5230, S5231, S524, S5240, S5241, S525, S5250, S5251, S526, S5260, S5261, S527, S5270, S5271, S528, S5280, S5281, S529, S5290, S5291, S720, S7200, S7201, S721, S7210, S7211, S722, S7220, S7221, S723, S7230, S7231, S724, S7240, S7241, S727, S7270, S7271, S728, S7280, S7281, S729, S7290, S7291, T021, T08, T0890
