# Supplementary material for: Polygenic risk score-guided personalized osteoporosis screening: a population-based study
Source: BMC Med. 2026 Jan 14;24:56. doi: 10.1186/s12916-025-04601-1 (PMC12849559; doi:10.1186/s12916-025-04601-1)
Supplement: Supplementary file 2 — Additional file 2: Figures S1–S7. Fig. S1-Age-specific 10-year cumulative risk curves in different PRS risk groups. Fig. S2 - The cumulative hazard of osteoporosis in different PRS risk groups at age 65. Fig. S3 - The cumulative hazard of osteoporosis in different PRS risk groups of all female participants. Fig. S4 - Cumulative hazard of osteoporosis in different PRS risk groups of postmenopausal women under 65 with low body weight. Fig. S5 - Age-specific 10-year cumulative risk curves in different PRS risk groups of postmenopausal women under 65 with low body weight. Fig. S6 - Flowchart of osteoporosis fracture risk as the primary outcome for women. Fig. S7 - Flowchart of osteoporosis fracture risk as the primary outcome for men. Table S1-S11. Table S1 - Age-specific 10-year cumulative risk (%) for women over 65 across different PRS risk groups. Table S2 - Age-specific 10-year cumulative risk (%) for women over 65 across different PRS risk groups, accounting for competing risks (loss to follow up and death). Table S3 - Hazard ratio (HR) and Risk advancement period (RAP) in different risk groups based on PRS (Excluding participants with missing covariates). Table S4 - Baseline characteristics for postmenopausal women under 65 with low body weight across different PRS risk groups. Table S5 - Age-specific 10-year cumulative risk (%) for postmenopausal women under 65 with low body weight across different PRS risk groups. Table S6 - Risk advancement period (RAP) and risk-adapted starting age of osteoporosis screening for postmenopausal women under 65 with low body weight across different PRS risk groups. Table S7 - Age-specific 10-year cumulative risk (%) of osteoporotic fracture for women. Table S8 - Age-specific 10-year cumulative risk (%) of osteoporotic fracture for men across different PRS risk groups. Table S9 - Risk advancement period (RAP) and risk-adapted starting age of osteoporotic fracture screening for women across different PRS risk groups. Table S10 - Ris [file 12916_2025_4601_MOESM2_ESM.docx]

## **Additional File 2**

**Fig. S1 Age-specific 10-year cumulative risk curves in different PRS risk groups.** Participants were divided into quartiles (Q1-lowest, Q2, Q3, and Q4-highest) based on the distributions of PRS in the present population. The orange line indicates the 10-year cumulative risk curve in the general female participants. The dashed line indicates the 10-year cumulative risk at age of 65 years old.


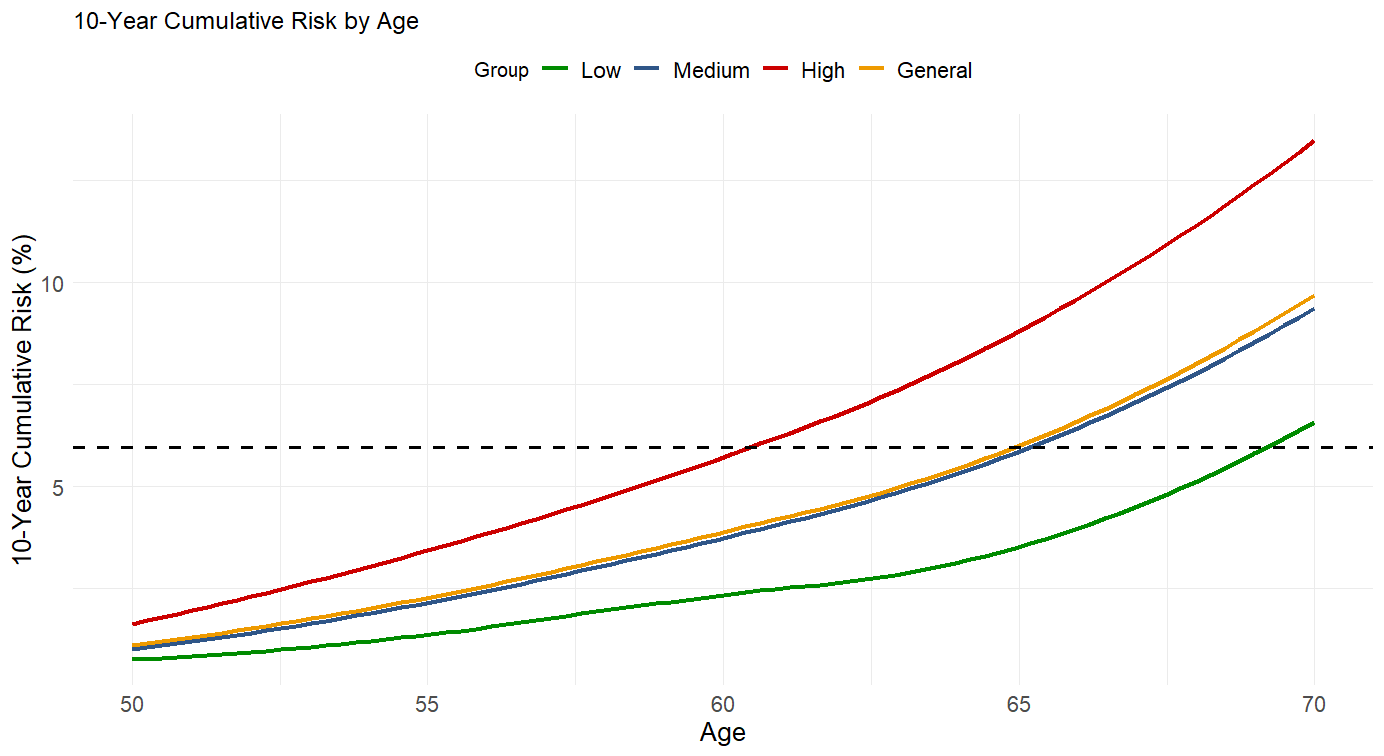


**Fig. S2 The cumulative hazard of osteoporosis in different PRS risk groups at age 65.** Participants were divided into quartiles (Q1-lowest, Q2, Q3, and Q4-highest) based on the distributions of PRS in female participants at 65 years old. The orange line indicates the 10-year cumulative hazard curve in the all 65-year-old female participants.


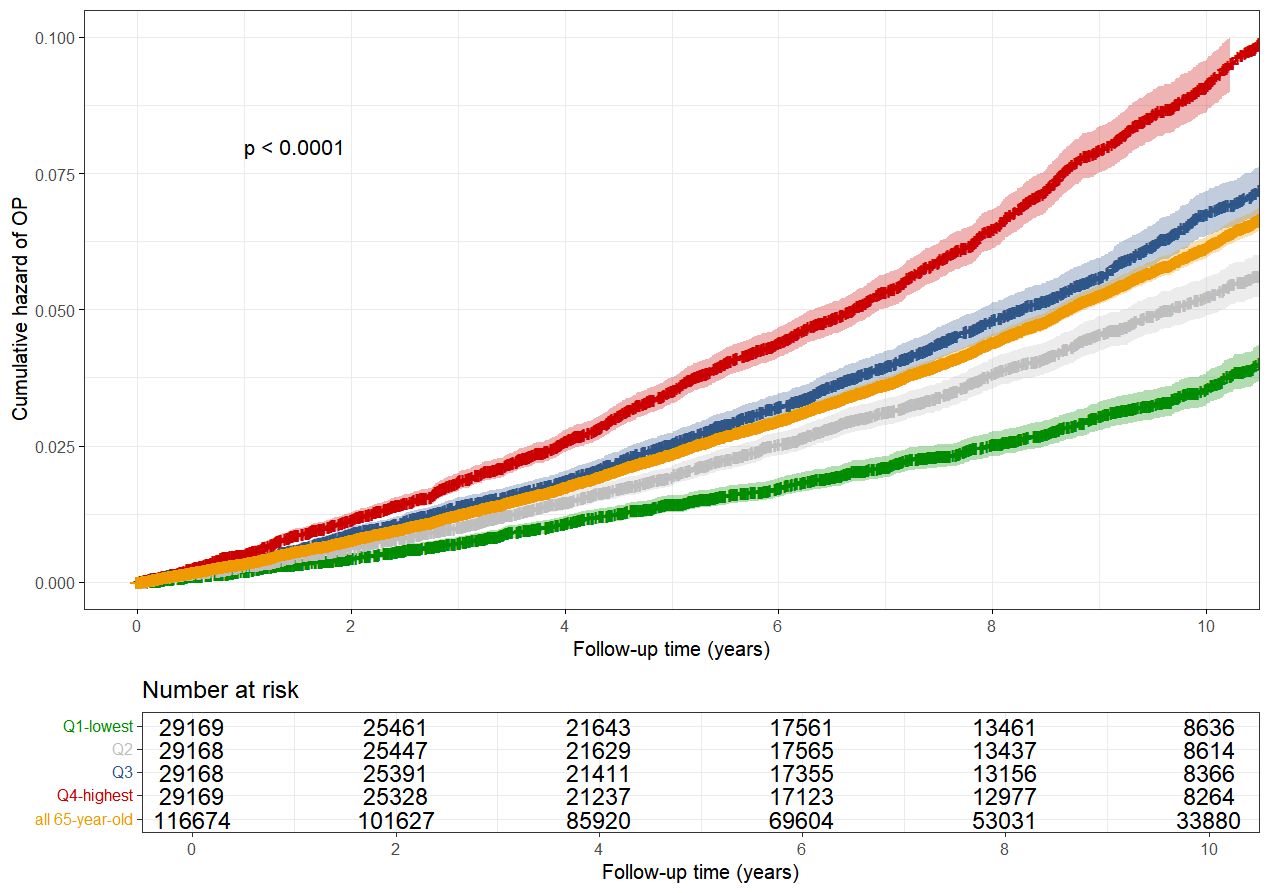


**Fig. S3 The cumulative hazard of osteoporosis in different PRS risk groups of all female participants.** Participants were divided into quartiles (Q1-lowest, Q2, Q3, and Q4-highest) based on the distributions of PRS in the present population. The orange line indicates the 10-year cumulative hazard curve in the general female participants.


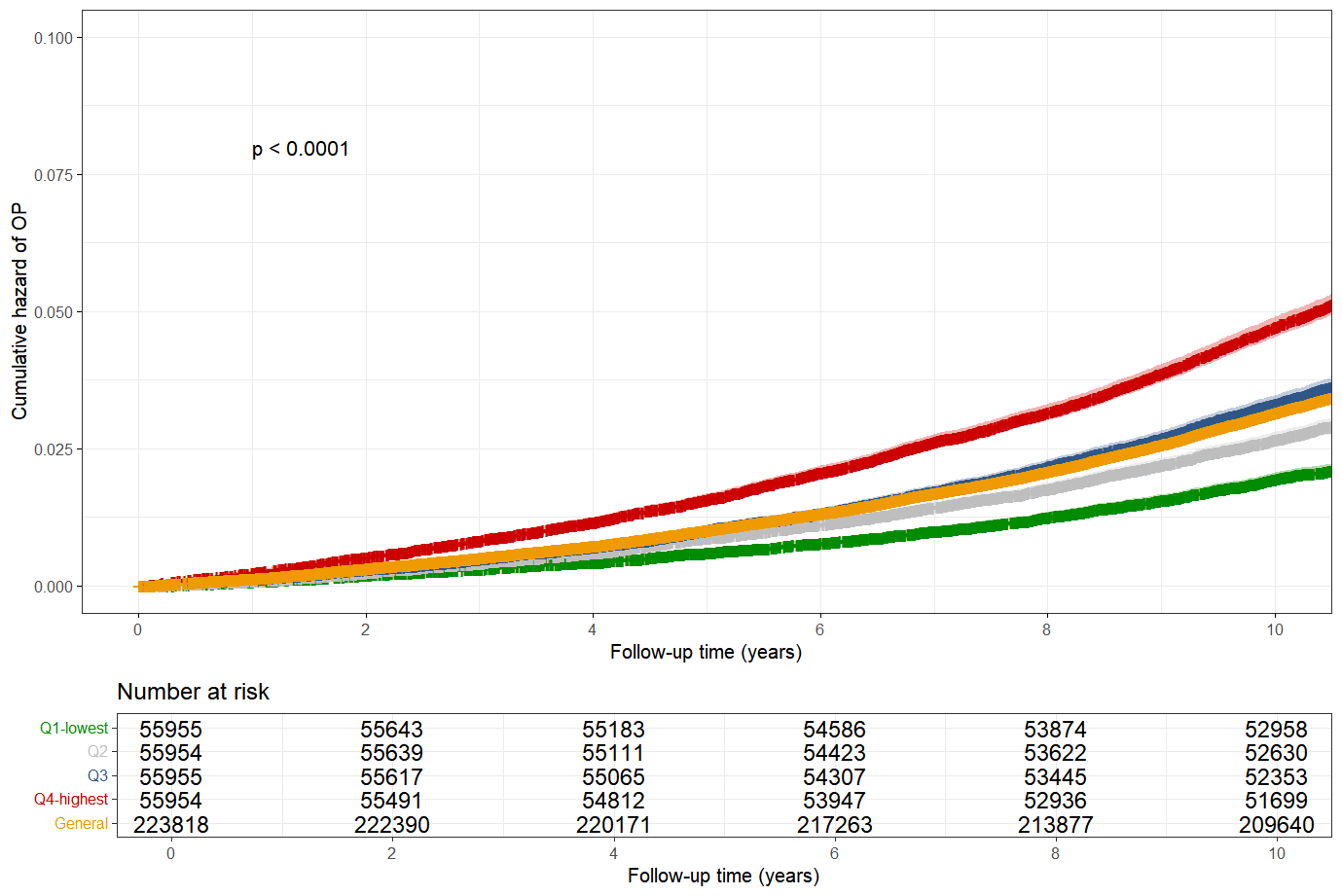


**Fig. S4 Cumulative hazard of osteoporosis in different PRS risk groups of postmenopausal women under 65 with low body weight.** Participants were divided into three risk groups (Low, Medium, and High) based on the distributions of PRS.


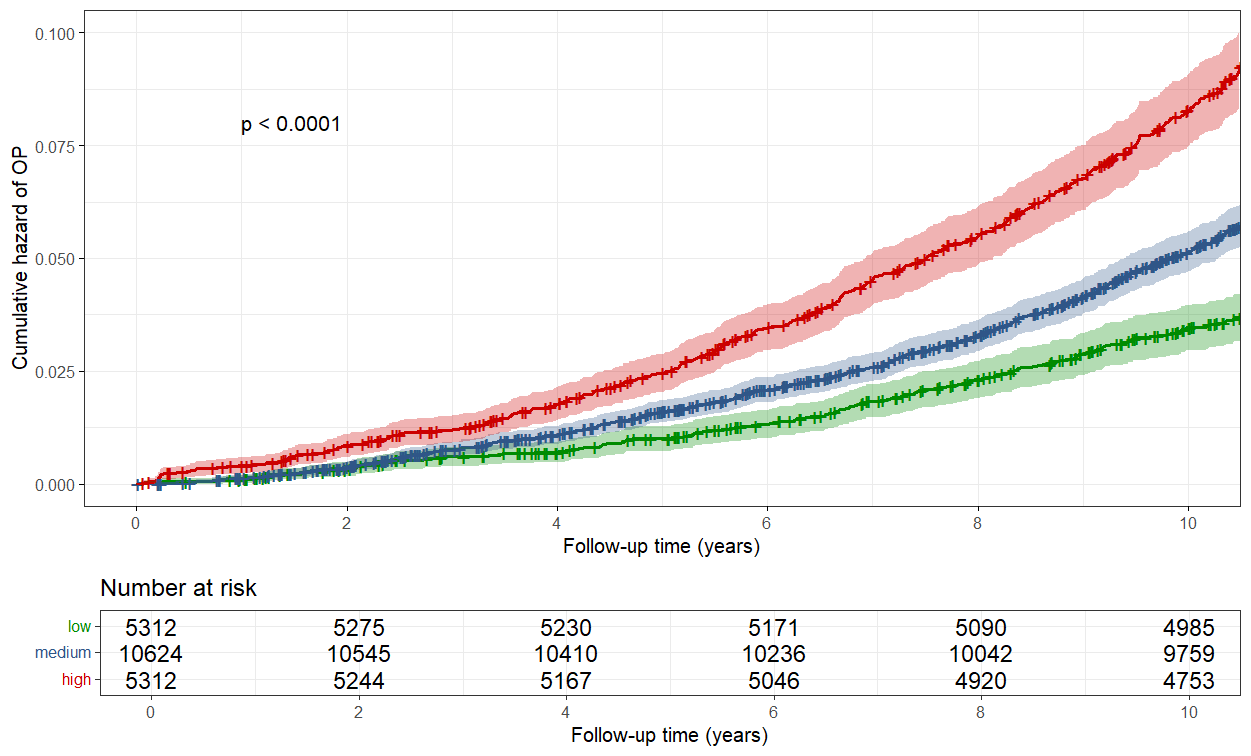


**Fig. S5 Age-specific 10-year cumulative risk curves in different PRS risk groups of postmenopausal women under 65 with low body weight.** Participants were divided into three groups (Low, Medium, and High) based on the distributions of PRS of postmenopausal women under 65 with low body weight. The dashed line indicates the 10-year cumulative risk at age of 65 years old (the starting age of screening for women older than 65 years old recommended by US Preventive Services Task Force) in the general female participants.


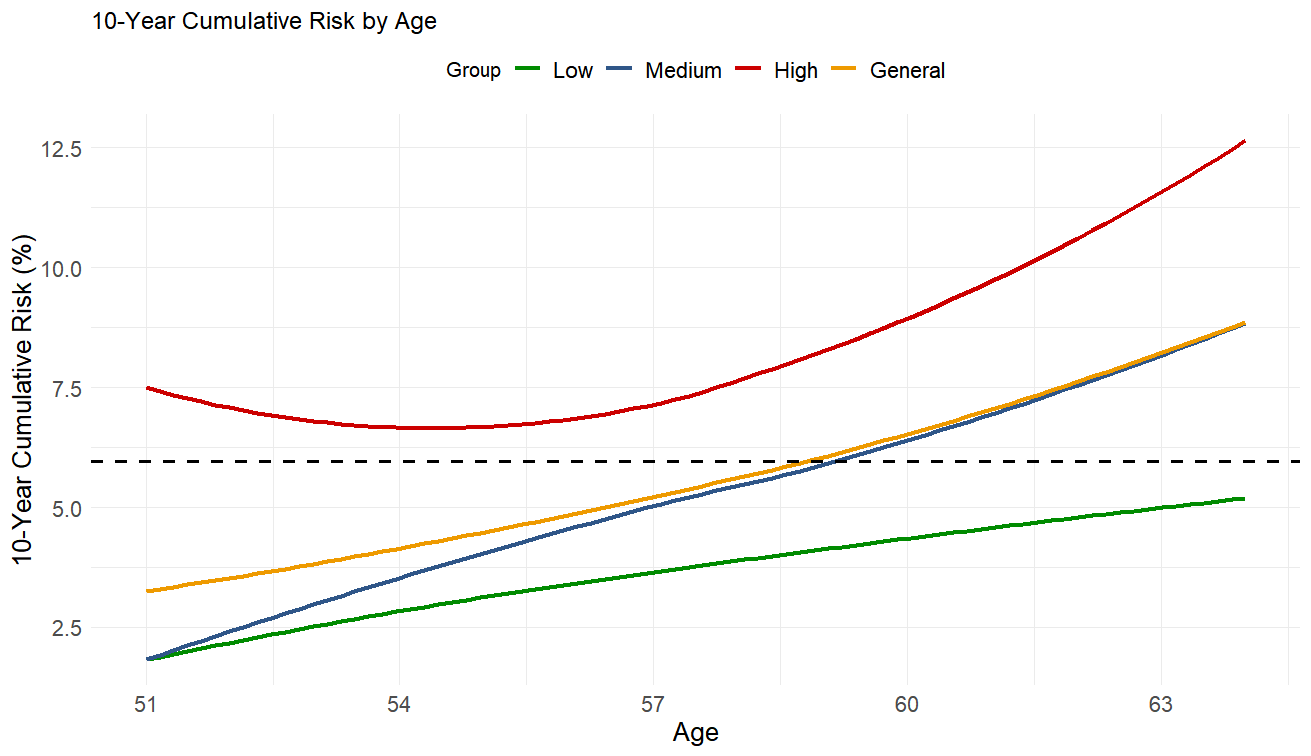


**Fig. S6 Flowchart of osteoporosis fracture risk as the primary outcome for women**


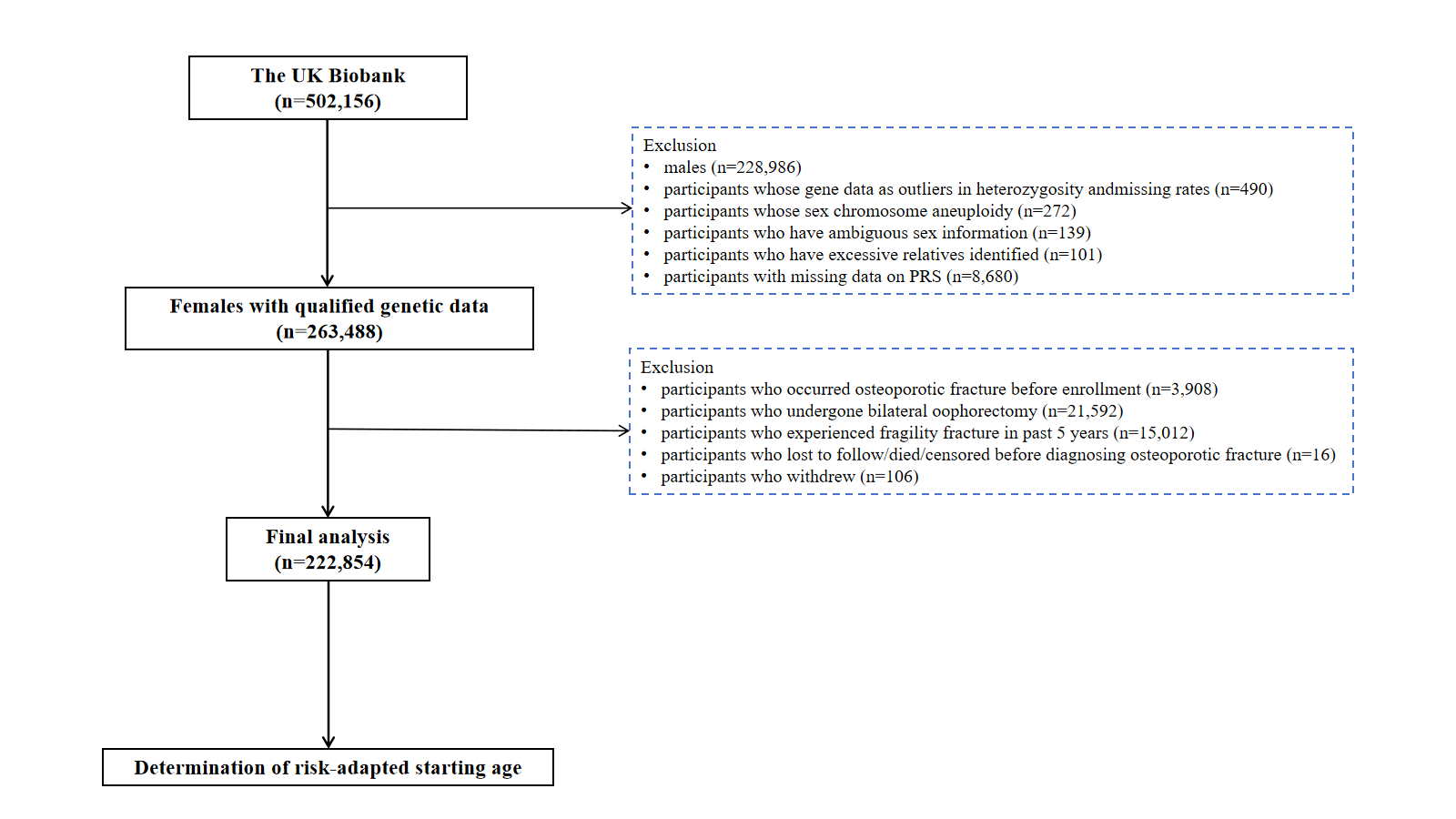


**Fig. S7 Flowchart of osteoporosis fracture risk as the primary outcome for men**


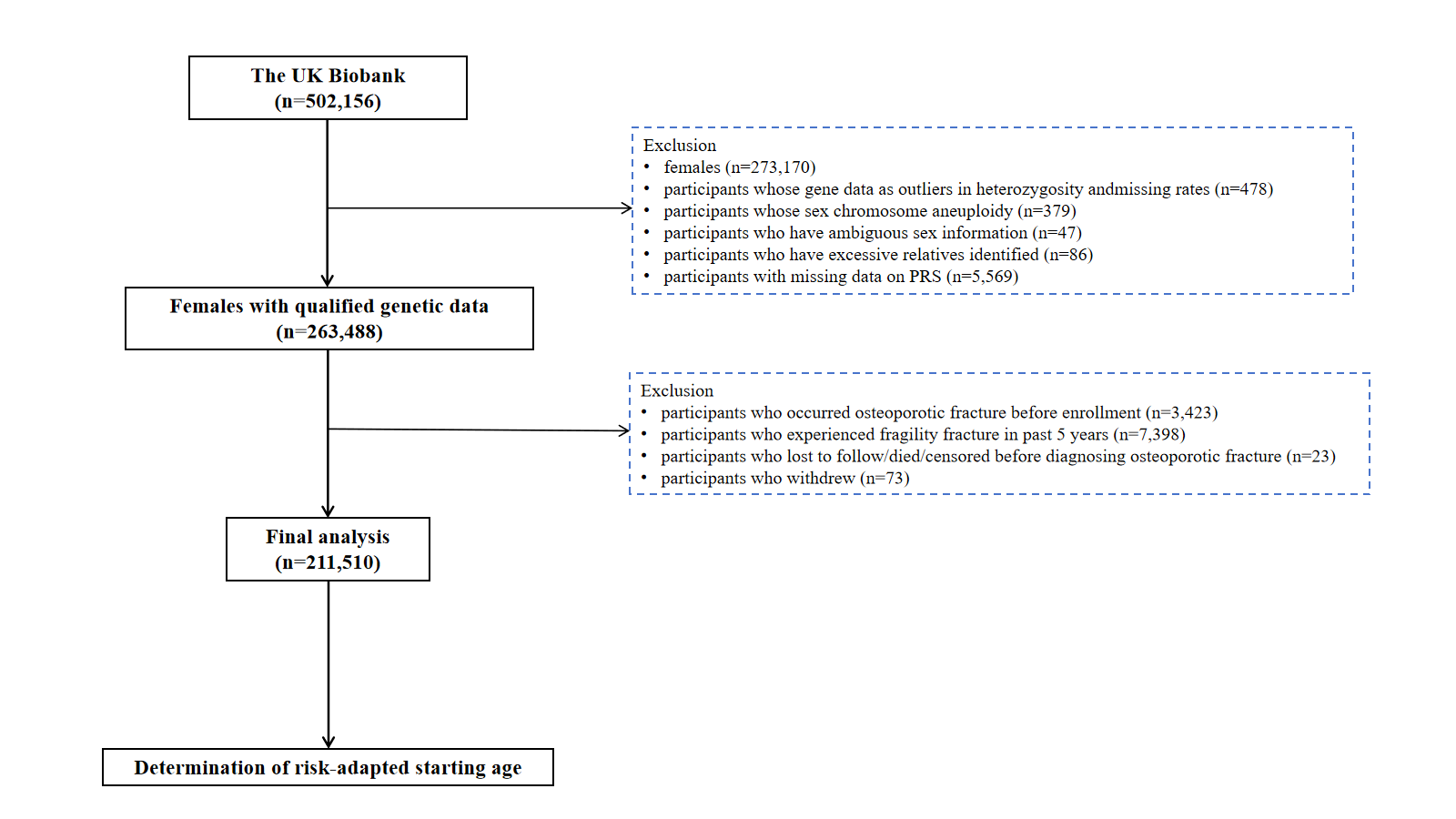


**Table S1. Age-specific 10-year cumulative risk (%) for women over 65 across different PRS risk groups.**

| Age | Low risk | Medium risk | High risk | General |
| --- | --- | --- | --- | --- |
| 50 | 0.75% | 1.02% | 1.64% | 1.11% |
| 51 | 0.80% | 1.18% | 1.86% | 1.26% |
| 52 | 1.03% | 1.42% | 2.34% | 1.56% |
| 53 | 1.06% | 1.64% | 2.71% | 1.77% |
| 54 | 1.13% | 1.88% | 2.98% | 1.97% |
| 55 | 1.29% | 2.10% | 3.60% | 2.27% |
| 56 | 1.53% | 2.34% | 3.75% | 2.49% |
| 57 | 1.65% | 2.81% | 4.19% | 2.86% |
| 58 | 2.00% | 3.14% | 4.72% | 3.25% |
| 59 | 2.25% | 3.37% | 5.24% | 3.56% |
| 60 | 2.43% | 3.72% | 5.76% | 3.90% |
| 61 | 2.50% | 4.12% | 6.39% | 4.28% |
| 62 | 2.59% | 4.41% | 6.67% | 4.52% |
| 63 | 2.84% | 4.88% | 7.31% | 4.97% |
| 64 | 3.10% | 5.33% | 8.16% | 5.47% |
| 65 | 3.48% | 5.85% | 8.70% | 5.95% |
| 66 | 3.94% | 6.43% | 9.62% | 6.59% |
| 67 | 4.45% | 7.00% | 10.53% | 7.23% |
| 68 | 5.10% | 7.65% | 11.36% | 7.93% |
| 69 | 5.92% | 8.65% | 12.42% | 8.90% |
| 70 | 6.55% | 9.38% | 13.49% | 9.69% |

**Table S2. Age-specific 10-year cumulative risk (%) for women over 65 across different PRS risk groups, accounting for competing risks (loss to follow up and death)**

| Age | Low risk | Medium risk | High risk | General |
| --- | --- | --- | --- | --- |
| 50 | 0.74% | 1.02% | 1.63% | 1.10% |
| 51 | 0.79% | 1.17% | 1.84% | 1.24% |
| 52 | 1.02% | 1.41% | 2.32% | 1.54% |
| 53 | 1.05% | 1.62% | 2.68% | 1.75% |
| 54 | 1.12% | 1.86% | 2.95% | 1.95% |
| 55 | 1.27% | 2.07% | 3.55% | 2.24% |
| 56 | 1.50% | 2.31% | 3.70% | 2.46% |
| 57 | 1.62% | 2.76% | 4.14% | 2.82% |
| 58 | 1.96% | 3.08% | 4.65% | 3.19% |
| 59 | 2.21% | 3.31% | 5.15% | 3.49% |
| 60 | 2.38% | 3.65% | 5.66% | 3.83% |
| 61 | 2.45% | 4.03% | 6.26% | 4.19% |
| 62 | 2.54% | 4.31% | 6.53% | 4.42% |
| 63 | 2.78% | 4.76% | 7.15% | 4.85% |
| 64 | 3.02% | 5.19% | 7.96% | 5.33% |
| 65 | 3.38% | 5.68% | 8.46% | 5.79% |
| 66 | 3.81% | 6.23% | 9.33% | 6.39% |
| 67 | 4.29% | 6.75% | 10.18% | 6.98% |
| 68 | 4.88% | 7.36% | 10.93% | 7.62% |
| 69 | 5.63% | 8.27% | 11.91% | 8.50% |
| 70 | 6.19% | 8.92% | 12.87% | 9.21% |

**Table S3. Hazard ratio (HR) and Risk advancement period (RAP) in different risk groups based on PRS (Excluding participants with missing covariates)**

|  | Case/Total (%) | HR^a^ (95%CI) | HR^b^ (95%CI) | RAP^b^ (95%CI) (years) |
| --- | --- | --- | --- | --- |
| PRS* | 6430/135220 (4.76) | 1.46 (1.43, 1.50) | 1.46 (1.42, 1.49) |  |
| Low | 952/33768 (2.82) | 0.62(0.57, 0.66) | 0.62 (0.57, 0.66) | -5.58 (-6.56, -4.82) |
| Medium | 3057/67668 (4.52) | Reference | Reference | Reference |
| High | 2421/33784 (7.17) | 1.63 (1.55, 1.72) | 1.62 (1.54, 1.71) | 5.62 (4.97, 6.33) |

*Associations of genetic risk (per standard deviation increment) with osteoporosis

a: model adjusted age and first 10 genetic principal components

b: model adjusted baseline age, ethnicity, body mass index (BMI), smoking status, alcohol consumption, physical activity, history of hormone replacement therapy (HRT) use, Townsend deprivation index (TDI), menopausal status, comorbidity count, history of parental hip fractures and the first 10 genetic principal components.

**Table S4. Baseline characteristics for postmenopausal women under 65 with low body weight across different PRS risk groups**

|  | Overall | Low risk | Moderate risk | High risk |
| --- | --- | --- | --- | --- |
| N | 21248 | 4999 | 10744 | 5505 |
| Follow time  (years, median (IQR)) | 13.59 [12.73, 14.28] | 13.63 [12.89, 14.29] | 13.58 [12.74, 14.28] | 13.55 [12.62, 14.29] |
| years, median (IQR)) | 59.00 [55.00, 62.00] | 59.00 [55.00, 62.00] | 59.00 [55.00, 62.00] | 59.00 [55.00, 62.00] |
| BMI (%) |  |  |  |  |
| <18.5 | 800 (3.8) | 193 (3.9) | 394 (3.7) | 213 (3.9) |
| 18.5-24.9 | 19389 (91.3) | 4564 (91.3) | 9804 (91.3) | 5021 (91.2) |
| 25-29.9 | 1048 (4.9) | 241 (4.8) | 539 (5.0) | 268 (4.9) |
| >= 30 | 7 (0.0) | 0 (0.0) | 4 (0.0) | 3 (0.1) |
| Missing | 4 (0.0) | 1 (0.0) | 3 (0.0) | 0 (0.0) |
| Smoking (%) |  |  |  |  |
| Never | 13148 (61.9) | 3069 (61.4) | 6627 (61.7) | 3452 (62.7) |
| Previous | 5924 (27.9) | 1407 (28.1) | 2981 (27.7) | 1536 (27.9) |
| Current | 2090 (9.8) | 496 (9.9) | 1095 (10.2) | 499 (9.1) |
| Missing | 86 (0.4) | 27 (0.5) | 41 (0.4) | 18 (0.3) |
| Alcohol consumption (%) |  |  |  |  |
| Never | 1210 (5.7) | 311 (6.2) | 572 (5.3) | 327 (5.9) |
| Former | 753 ( 3.5) | 177 (3.5) | 363 (3.4) | 213 (3.9) |
| Occasional | 1839 (8.7) | 470 (9.4) | 909 (8.5) | 460 (8.4) |
| Moderate | 9719 (45.7) | 2246 ( 44.9) | 4995 ( 46.5) | 2478 (45.0) |
| Increasing risk | 4014 (18.9) | 924 (18.5) | 2040 (19.0) | 1050 (19.1) |
| High risk | 439 (2.1) | 111 (2.2) | 209 (1.9) | 119 (2.2) |
| Missing | 3274 (15.4) | 760 (15.2) | 1656 (15.4) | 858 (15.6) |
| Morbidity count (%) |  |  |  |  |
| 0 | 9213 (43.4) | 2187 (43.7) | 4630 (43.1) | 2396 (43.5) |
| 1-3 | 11496 (54.1) | 2686 (53.7) | 5851 (54.5) | 2959 (53.8) |
| >=4 | 539 (2.5) | 126 (2.5) | 263 (2.4) | 150 (2.7) |
| HRT use history (%) |  |  |  |  |
| Yes | 12662 (59.6) | 2987 (59.8) | 6402 (59.6) | 3273 (59.5) |
| No | 8498 (40.0) | 1993 (39.9) | 4297 (40.0) | 2208 (40.1) |
| Missing | 88 (0.4) | 19 (0.4) | 45 (0.4) | 24 (0.4) |
| Age of menopause (years, median (IQR)) | 51.00 [49.00, 53.00] | 51.00 [49.00, 53.00] | 51.00 [49.00, 53.00] | 51.00 [49.00, 53.00] |
| Physical activity (median [IQR]) | 2013.00  [975.00, 3843.00] | 2014.00  [985.12, 3849.00] | 2008.50  [968.50, 3813.00] | 2019.00  [990.00, 3906.00] |

**Table S5. Age-specific 10-year cumulative risk (%) for postmenopausal women under 65 with low body weight across different PRS risk groups**

| Age | Low risk | Medium risk | High risk | General |
| --- | --- | --- | --- | --- |
| 50 | 1.60% | 2.58% | 7.57% | 3.55% |
| 51 | 1.36% | 1.90% | 7.16% | 3.08% |
| 52 | 2.94% | 2.25% | 7.60% | 3.78% |
| 53 | 2.56% | 2.92% | 7.09% | 3.89% |
| 54 | 2.51% | 3.59% | 6.02% | 3.92% |
| 55 | 3.07% | 4.25% | 7.10% | 4.67% |
| 56 | 3.44% | 4.47% | 6.31% | 4.67% |
| 57 | 3.56% | 5.31% | 6.99% | 5.30% |
| 58 | 3.90% | 5.54% | 7.99% | 5.75% |
| 59 | 4.49% | 5.67% | 8.28% | 6.04% |
| 60 | 4.35% | 6.06% | 8.85% | 6.34% |
| 61 | 4.32% | 6.89% | 10.38% | 7.14% |
| 62 | 4.59% | 7.34% | 10.50% | 7.44% |
| 63 | 5.09% | 8.14% | 11.32% | 8.16% |
| 64 | 5.27% | 9.09% | 12.64% | 9.00% |

**Table S6. Risk advancement period (RAP) and risk-adapted starting age of** **osteoporosis screening for postmenopausal women under 65 with low body weight across different PRS risk groups**

|  | Case/Total (%) | HR^a^ (95%CI) | HR^b^ (95%CI) | RAP^b^ (95%CI) (years) | Risk-adapted starting age (years) of screening |
| --- | --- | --- | --- | --- | --- |
| PRS* | 1850/21248 (8.7) | 1.43 (1.37,1.51) | 1.41 (1.34,1.47) |  |  |
| Low | 296/5312 (5.6) | 0.64 (0.56, 0.72) | 0.62 (0.54, 0.71) | -6.57 (-8.87, -4.44) | >64 |
| Medium | 905/10624 (8.5) | Reference | Reference | Reference | 59 |
| High | 649/5312 (12.2) | 1.49 (1.35, 1.65) | 1.47 (1.33, 1.63) | 5.37 (3.73, 7.06) | <54 |

*Associations of genetic risk (per standard deviation increment) with osteoporosis

a: model adjusted age and first 10 genetic principal components

b: model adjusted baseline age, ethnicity, body mass index (BMI), smoking status, alcohol consumption, physical activity, history of hormone replacement therapy (HRT) use, Townsend deprivation index (TDI), comorbidity count, history of parental hip fractures and the first 10 genetic principal components.

**Table S7. Age-specific 10-year cumulative risk (%) of osteoporotic fracture for women**

| Age | Low risk | Medium risk | High risk | General |
| --- | --- | --- | --- | --- |
| 50 | 1.46% | 2.13% | 2.69% | 2.11% |
| 51 | 1.54% | 2.29% | 3.05% | 2.30% |
| 52 | 1.69% | 2.59% | 3.35% | 2.56% |
| 53 | 1.77% | 2.70% | 3.56% | 2.69% |
| 54 | 1.87% | 2.91% | 3.80% | 2.88% |
| 55 | 2.06% | 3.09% | 4.13% | 3.09% |
| 56 | 2.02% | 3.15% | 4.09% | 3.10% |
| 57 | 2.27% | 3.28% | 4.22% | 3.27% |
| 58 | 2.30% | 3.33% | 4.39% | 3.34% |
| 59 | 2.62% | 3.56% | 4.68% | 3.60% |
| 60 | 2.76% | 3.77% | 5.01% | 3.83% |
| 61 | 3.08% | 4.17% | 5.25% | 4.16% |
| 62 | 3.28% | 4.26% | 5.55% | 4.33% |
| 63 | 3.40% | 4.58% | 5.91% | 4.62% |
| 64 | 3.52% | 4.87% | 6.26% | 4.87% |
| 65 | 3.77% | 5.20% | 6.75% | 5.22% |
| 66 | 4.08% | 5.59% | 7.29% | 5.63% |
| 67 | 4.53% | 6.14% | 7.89% | 6.17% |
| 68 | 5.13% | 6.77% | 8.47% | 6.78% |
| 69 | 5.49% | 7.61% | 9.45% | 7.53% |
| 70 | 6.11% | 8.27% | 10.45% | 8.27% |

**Table S8. Age-specific 10-year cumulative risk (%) of osteoporotic fracture for men across different PRS risk groups**

| Age | Low risk | Medium risk | High risk | General |
| --- | --- | --- | --- | --- |
| 50 | 1.39% | 1.80% | 2.12% | 1.78% |
| 51 | 1.47% | 1.75% | 2.21% | 1.79% |
| 52 | 1.56% | 1.73% | 2.42% | 1.86% |
| 53 | 1.36% | 1.86% | 2.43% | 1.88% |
| 54 | 1.37% | 1.96% | 2.57% | 1.97% |
| 55 | 1.56% | 1.94% | 2.52% | 1.99% |
| 56 | 1.71% | 2.03% | 2.65% | 2.10% |
| 57 | 1.80% | 2.18% | 2.89% | 2.26% |
| 58 | 1.87% | 2.14% | 2.97% | 2.28% |
| 59 | 1.89% | 2.22% | 3.01% | 2.33% |
| 60 | 1.93% | 2.30% | 3.28% | 2.45% |
| 61 | 1.97% | 2.34% | 3.23% | 2.47% |
| 62 | 1.96% | 2.42% | 3.28% | 2.52% |
| 63 | 2.05% | 2.56% | 3.23% | 2.60% |
| 64 | 2.06% | 2.63% | 3.34% | 2.67% |
| 65 | 2.24% | 2.84% | 3.65% | 2.89% |
| 66 | 2.51% | 3.25% | 3.88% | 3.22% |
| 67 | 2.65% | 3.63% | 4.28% | 3.55% |
| 68 | 2.91% | 3.96% | 4.98% | 3.95% |
| 69 | 3.14% | 4.42% | 5.42% | 4.35% |
| 70 | 3.55% | 4.90% | 5.86% | 4.80% |

**Table S9. Risk advancement period (RAP) and risk-adapted starting age of osteoporotic fracture screening for women across different PRS risk groups**

|  | Case/Total (%) | HR (95%CI) | RAP (95%CI) (years) | Risk-adapted starting age (years) of screening |
| --- | --- | --- | --- | --- |
| PRS* | 12026/222854 (5.4) | 1.26 (1.24, 1.29) |  |  |
| Low | 2195/55714 (3.9) | 0.74 (0.71, 0.78) | -4.28 (-5.04, -3.61) | 68 |
| Medium | 5925/111426 (5.3) | Reference | Reference | 65 |
| High | 3906/55714 (7.0) | 1.34 (1.29, 1.39) | 4.16 (3.61, 4.74) | 61 |

*Associations of genetic risk (per standard deviation increment) with osteoporosis

Model adjusted age and first 10 genetic principal components

**Table S10. Risk advancement period (RAP) of osteoporotic fracture for men across different PRS risk groups**

|  | Case/Total (%) | HR (95%CI) | RAP (95%CI) (years) |
| --- | --- | --- | --- |
| PRS* | 7317/211510 (3.5) | 1.22 (1.20, 1.25) |  |
| Low | 1393/52878 (2.6) | 0.77 (0.73, 0.82) | -5.82 (-7.24, -4.47) |
| Medium | 3617/105754 (3.4) | Reference | Reference |
| High | 2307/52878 (4.4) | 1.29 (1.22, 1.36) | 5.71 (4.50, 6.88) |

*Associations of genetic risk (per standard deviation increment) with osteoporosis

Model adjusted age and first 10 genetic principal components

**Table S11. Risk advancement period (RAP) and risk-adapted starting age of OP screening in different risk groups based on PRS using flexible parametric survival models**

|  | Case/Total (%) | HR^a^ (95%CI) | HR^b^ (95%CI) | RAP^b^ (95%CI) (years) |
| --- | --- | --- | --- | --- |
| PRS* | 11639/223818(5.2) | 1.41 (1.38, 1.43) | 1.40 (1.38, 1.43) |  |
| Low | 1848/55955(3.3) | 0.66 (0.62, 0.69) | 0.66 (0.62, 0.69) | -4.89 (-6.54, -4.69) |
| Medium | 5585/111909(5.0) | Reference | Reference | Reference |
| High | 4206/55954(7.5) | 1.55 (1.49, 1.61) | 1.54 (1.48, 1.60) | 4.99 (4.94, 6.28) |
